# Poor sleep quality is linked to increased frailty in middle-aged people living with HIV in Botswana

**DOI:** 10.21203/rs.3.rs-4462187/v1

**Published:** 2024-06-07

**Authors:** Xi Zheng, Ruixue Cai, Chenlu Gao, Ponego Ponatshego, Lei Gao, Monty A. Montano, Kun Hu, Mosepele Mosepele, Peng Li

**Affiliations:** Massachusetts General Hospital; Massachusetts General Hospital; Massachusetts General Hospital; University of Botswana; Massachusetts General Hospital; Brigham and Women’s Hospital; Massachusetts General Hospital; University of Botswana; Massachusetts General Hospital

**Keywords:** Multi-morbidity, holistic health, aging, HIV comorbidities

## Abstract

This work aims to evaluate associations between self-reported sleep health and frailty in Botswana, a sub-Saharan Africa setting. Fifty persons living with HIV (PLWH) on suppressive antiretroviral therapy (ART) and fifty HIV seronegative control participants are enrolled in Botswana. Sleep quality is scored subjectively as “good” or “poor” based on self-report. A frailty index (FI) is constructed based on thirty-three health deficits related to body mass index, waist circumference, physical activity, emotional status, and fatigue, and scored ranging between 0 (no deficit present) and 1 (all deficits present). Sleep quality between PLWH and controls is compared using logistic regression; linear regression is performed to compare the FI between them. Linear regressions are performed to examine the association between the FI and sleep quality stratified by HIV serostatus. Age, sex, and comorbidities are adjusted; when relevant, CD4 cell and ART duration are controlled. PLWH display 2.88 (95% CI: 1.22–6.79, p = 0.02) higher odds of having poor sleep than controls. Having poor sleep is associated with increased FI in PLWH but not in controls. Specifically, compared with PLWH who have good sleep, PLWH who report poor sleep have a > 1 standard deviation (p < 0.0001) increase in their FI score.

## Introduction

People living with HIV infection (PLWH) face more challenges than HIV seronegative people in striving for healthy aging with a disproportionate burdened with multi-morbidities and geriatric syndromes,^1,2^ such as frailty.^3^ Frailty, a condition of reduced physiological reserve and vulnerability to stressors, is characterized clinically as either a physiologic condition (i.e., as having three or more of the following components: unexplained weight loss, weakness, exhaustion, slow walk speed, and low physical activity^4^) or as an accumulation of health deficits, commonly including approximately 30 items.^5^ Identifying frailty and the relevant causes or correlates at early stages may help advance treatment options and promote successful aging. In a previously matched controlled study, the prevalence of frailty in PLWH was reported to be three times greater than that in matched seronegative people.^6^ The onset of frailty was also found to occur at earlier ages in PLWH than in the general population.^7^ Mid-life risk factors predisposing PLWH to higher frailty risk remain poorly understood.

Sleep disturbances affect health homeostasis,^8^ and may adversely increase the risk for frailty, as evidenced by previous observational studies.^9–12^ Also, subjective and objective sleep measures are independently associated with frailty in older adults.^9,13^ Sleep disturbances, such as poorer sleep quality, inappropriate sleep timing and quantity, and sleep disorders such as insomnia and obstructive sleep apnea syndrome, are disproportionate in PLWH, affecting up to 70% of this population,^14^ twice the general population.^15^ However, potential associations between sleep disturbances and frailty in PLWH are unclear,^16^ especially in resource-limited settings such as Africa (and parts of the US), where the endeavor towards successful aging confronts more challenges due to structural inequities and disparities in healthcare as well as non-health sectors (i.e., social environmental determinants).^17^

This pilot study aimed to evaluate self-reported sleep disturbances in PLWH and relevance to frailty in a high HIV-prevalence setting within Africa. The study mainly involved analysis of data previously collected in a human study conducted in Botswana, sub-Saharan Africa, in which participants self-reported data related to their sleep behaviors. Other relevant clinical information was obtained during the study visit from self-report, examinations, and available medical records.

## Results

Table 1 summarizes the basic and clinical characteristics of all participants as obtained from patient self-reports and available medical records at the participating health facility.

PLWH showed worse sleep quality than HIV seronegative controls (Table 2). Specifically, after adjusting for age, sex, and the burden of chronic disease, PLWH showed a 2.88-fold increase in the odds of having poor sleep quality (95% CI: 1.22–6.79, *p* = 0.02) compared with seronegative controls. Based on a three-level sleep quality scoring approach (i.e., good, worse, worst), the observations were consistent. Specifically, PLWH showed a 1.69-fold (95% CI:1.06–2.75, *p* = 0.03) increase in the odds of having worse sleep quality and a 1.76-fold (95% CI: 0.95–3.45, *p* = 0.08) increase in the odds of having worst sleep quality than seronegative controls.

A score for frailty index (FI) did not differ significantly between PLWH and controls. After adjusting for age, sex, and the burden of chronic diseases, the FI score was 0.19 (95% CI: 0.15–0.23) in the control group. The FI in PLWH was 0.01 greater (95% CI: −0.01–0.04), which was not statistically signi cant (*p* = 0.38).

However, poor sleep quality was associated with a more serious frailty status in PLWH (Table 3). Specifically, after adjusting for age, sex, and the burden of chronic disease, the FI score was 0.14 (95% CI: 0.08–0.20) in those who had good sleep quality, whereas a 0.15 (95% CI: 0.09–0.21, *p* < 0.0001) increase in the FI was observed in those having poor sleep quality. In the fully adjusted model, poor sleep quality was associated with a 0.14 (95% CI: 0.07–0.21, *p* = 0.0004) increase in the FI in PLWH. Using a three-level sleep quality approach, we found that the largest values of the FI occurred in the PLWH whose sleep quality was the worst (i.e., compared with good: estimate = 0.21, 95% CI: 0.12–0.30, *p* < 0.0001], then in participants whose sleep quality was worse (i.e., compared with good: estimate = 0.12, 95% CI: 0.06–0.19, *p* = 0.0004. The change in the FI appeared monotonic as having the worst sleep quality was associated with an increase of 0.09 in the FI (95% CI: 0.00–0.17, *p* = 0.05) compared with those having worse sleep quality. These observations remained true after further adjusting for CD4 cell counts and ART duration.

In matched HIV seronegative controls, the association between sleep and frailty was not statistically significant when using the dichotomous sleep quality variable. When using the three-level sleep quality score, however, there was a statistically significant difference in the FI when comparing those with the worst sleep quality with those with good sleep quality (estimate = 0.15, 95% CI: 0.03–0.27, *p* = 0.01).

## Discussion

It is increasingly recognized that sleep disturbance can increase risk for impaired cognitive and physical functions in the general population. Borrowing the idea of an accumulation of health deficits, we established a continuous index based on physical assessments or questionnaires for physical frailty and studied potential correlates of frailty during mid-life in terms of sleep health in PLWH. We found that PLWH had worse self-reported sleep than controls, and that worse sleep quality in PLWH was linked to an increased burden of physical frailty in PLWH.

Our results that PLWH had poorer sleep health are consistent with prior research. For example, in a large cohort study conducted in PLWH in French, PLWH experienced 2.66-fold higher risk of insomnia.^18^ In a cohort study in Ethiopia, the prevalence of having difficulty maintaining sleep was over 50% in PLWH.^19^ While both the two previous studies also linked insomnia or poorer sleep quality with mental health outcomes such as anxiety and depression, our current research adds to this exiting knowledge by demonstrating that sleep quality may contribute to physical frailty in PLWH.

Mechanisms underlying the association between sleep disturbance and frailty in PLWH remain unclear and are yet to be determined. Potential candidates may include HIV-associated accelerated/accentuated biological aging and bioenergetic dysfunction that pose greater vulnerability to frailty when sleep is disturbed. For example, HIV infection can cause epigenetic modifications, including genes that regulate inflammation, speeding up the biological aging process in PLWH. HIV infection upregulates methyltransferase, resulting in methylation of CpG sites in DNA,^20–22^ which is closely related to biological aging. Additionally, PLWH often are socioeconomically disadvantaged, which can also lead to epigenetic modifications.^23–25^ In our recent studies, reduced nicotinamide adenine dinucleotide levels were found in skeletal muscles of PLWH,^26^ implying dysregulation in bioenergetic that leads to a physically vulnerable state to frailty. Other potential pathways may include inflammatory and coagulator responses, oxidative stress, autonomic control, and atherosclerosis^27^, which are all modulated by sleep. Future studies, ideally based on systems biology approaches, are warranted to systematically investigate these pathways that potentially involve multiple biological and physiological processes.

Sleep disorders such as insomnia can be symptoms of depression and anxiety; they may also contribute to their onset and progress.^28^ In the short term, neurobiological processes during sleep influence the next day’s mood^29^ and energy level.^30^ Over time, abnormal sleep quantity (i.e., too little or too much sleep), poor sleep quality, and sleep disorders adversely influence physiologic biomarkers (e.g., inflammation)^31^ and contribute to deficits in brain health.^32^

Additionally, prior model-based and human studies have suggested the importance of the circadian clock^33^ in health homeostasis. Circadian optimized interventions, such as dietary restriction and time-restricted feeding,^34^ also become attractive and promising methods for mitigating age-related physical decline and chronic diseases.^35^ The circadian system is a known critical regulator of sleep, and circadian disturbances may contribute to oxidative stress, cellular dysfunction, and neuroinflammation in disease states,^36,37^ all of which is widely involved in sleep homeostasis. Therefore, future studies in PLWH should consider assessing both sleep and circadian health and examine their roles in holistic cognitive and physical health outcomes in PLWH.

Several limitations should be considered when interpreting the results of this study. Firstly, as a pilot study, the sample size is relatively small, which restricts the generalizability of the results to a broader population. Secondly, as a cross-sectional study, the lack of follow-up on these subjects limits the capacity to understand the underlying causal directions. Lastly, the reliance on routine care data may also pose a potential limitation in verifying the accuracy and completeness of the recorded comorbidities. Despite these limitations, this pilot study could still contribute to the understanding of sleep health disparities as a potential physiological driver for frailty in PLWH in an Africa setting. Our findings promote an awareness of sleep health and clinical relevance for frailty risk, especially in PLWH.

## Methods

### Study design and participants

This pilot study involved analysis of data collected from a previous study conducted in a large village on the outskirts of the Gaborone (capital city), Botswana, sub-Saharan Africa. The study was approved by the University of Botswana Human Research Ethics Committee and the Botswana Ministry of Health Human Research Ethics Committee (HRDC) and was performed in accordance with the ethical standards laid down in the 1964 Declaration of Helsinki and its later amendments. All participants provided written informed consent. In total, 100 participants were recruited between March and December 2021, and they completed extensive investigator developed questionnaires on demographics, weight loss, obesity, physical activity, emotion, and fatigue. Among them, 50 participants were HIV seropositive (i.e., PLWH) and on suppressive antiretroviral therapy (ART) regimens; 50 participants were confirmed seronegative who were matched with PLWH with age, sex, and common chronic disease histories.

### Assessment of Sleep

Participants self-reported their sleep behaviors by answering two questions related to sleep quality: (1) “I sleep well” and (2) “I need to sleep during the day.” For both questions, the response options include “Not at all”, “A little bit”, “Somewhat”, “Quite a bit”, and “Very much”. Participants were scored “1 (i.e., good sleep quality)” if they answered “Very much” to question (1) and “Not at all” to question (2), and were scored “0 (i.e., poor sleep quality)” if otherwise. Additionally, we employed an ordinal three-level scoring scheme: participants who answered “very much” to question (1) and “not at all” to question (2) were given a score 2 (i.e., good sleep quality); participants who answered “very much” to question (1) or “not at all” to question (2) were given a score 1 (i.e., worse sleep quality); and a score 0 was given to those who chose other answers (i.e., worst sleep quality).

### Assessment of Frailty

We used the accumulation of health deficits approach to construct a continuous index for frailty (frailty index, or FI).^38^ In total, 33 health deficits across four health domains [i.e., anthropometric measures, physical activity level, emotional status, and fatigue] were assessed based on physical measurements or self-reports from questionnaires. Anthropometric measures included body mass index (BMI) and waist circumference. BMI and waist circumference together can help identify a high-risk obesity phenotype.^39^ BMI is measured from weight and height.^40^ Participants with BMI < 18.5 (i.e., underweight) or > = 25 (i.e., overweight) were assigned a score “1”, whereas participants whose BMI fell within [18.5, 25) were assigned a score “0”.^40^ The measurement of waist circumference is directly related to the amount of abdominal fat, which serves as an indicator of abdominal obesity.^40^ For waist circumference, we applied sex-stratified criteria for scoring: a score “1” was given to women with a waist circumference > 88 cm and men with a waist circumference > 102 cm, and a score “0” was given if otherwise.^40^ Physical activity level was assessed based on 12 questions from the Duke Activity Status Index (DASI).^41^ Emotional status was assessed using 6 questions from the “Emotional well-being” part of the Functional Assessment of Chronic Illness Therapy – Spiritual Well-Being (FACIT-Sp) measurement system.^42^ Fatigue symptoms were assessed using 13 questions from the Functional Assessment of Chronic Illness Therapy – Fatigue (FACIT-F).^43^ **Table S1** summarizes each question and the scoring criteria for each item of the last three health domains. The final frailty index was expressed as a ratio of deficits present in all the above-mentioned 33 heath deficits. Therefore, the continuous frailty index ranged between 0 and 1; a higher score means more frail.

### Assessment of Covariates

We assessed participants’ medical history through a combination of self-reported questionnaires or measurements during the visit. Covariates included demographics (age and sex), chronic comorbidities, as well as duration of ART and CD4 cell count (for PLWH only). Age at visit of assessment was calculated in years based on their dates of birth. Sex (male/female) was self-reported. Participants also self-reported the presence/absence of chronic diseases (and these were verified from their medical records) including diabetes mellitus, liver disease, malignancy, chronic kidney disease, bone marrow transplant, hemodialysis, solid organ transplant, neurological disease, steroid use, cardiovascular disease, respiratory disease, hepatitis, and hypertension/high blood pressure. The burden of chronic comorbidities (yes/no) divided participants into two groups, where “yes” indicated the group with at least one disease and “no” represented the group free from these chronic conditions. The duration that PLWH had been on ART was calculated as the time lag between the date of ART initiation to the date of study visit in years. Both viral load and CD4 count were also abstracted from the medical records (obtained as part of standard of care).

### Statistical Analysis

Descriptive characteristics are presented as means with standard deviations (SDs) for quantitative variables (normally distributed) and medians with interquartile ranges (nonnormally distributed). Categorical variables are presented as percentages. To examine the disparity in sleep health between PLWH and controls, a logistic regression model was conducted with the group (i.e., PLWH or controls) as a predictor and with the sleep quality variable (good or poor) as a dichotomous outcome. Alternatively, a nominal logistic regression model was conducted with the group as a predictor and with the three-level sleep quality (i.e., good, worse, or worst) as an ordinal outcome variable. A linear regression model was performed to examine the disparity in frailty between PLWH and controls, with the group as a predictor and the FI as a continuous outcome. These models were adjusted for age, sex, and the burden of chronic comorbidities. Linear regression models for the continuous FI score stratified by group were performed to examine the association between sleep and frailty. The dichotomous sleep quality variable (good or poor) was included as a predictor. The three-level sleep quality variable was used as a nominal predictor in a separate series of models. Age, sex, and the burden of chronic comorbidities were included as covariates. Additionally, CD4 cell counts and ART duration were further included as covariates in the model for PLWH. All statistical analyses were performed using JMP Pro (Ver. 16, SAS Institute, Cary, NC, USA). Double-sided *p* < 0.05 was used for statistical significance.

## Figures and Tables

**Table 1.** Demographics and clinical characteristics of participants.

	PLWH	Controls	*p* value
	(n = 50)	(n = 50)	
Age (year)	56.9 (4.4)	56.6 (4.3)	0.80
Female Sex	26 (52%)	24 (48%)	0.84
Comorbidities Burden			
Diabetes Mellitus	2 (4%)	3 (6%)	-
Hypertension	13 (26%)	14 (28%)	-
Any of above	14 (28%)	15 (30%)	0.83
Waist Circumference (cm)	89.97 (12.44)	94.08 (12.51)	0.10
BMI (kg/m^2^)	25.13 (6.4)	27.94 (5.24)	0.02
ART Duration (years)	11.04 (5.29)	-	-
CD4 Count (cells/ul)	719.64 (267.63)	-	-
Sleep (dichotomous)			0.04
Good	22 (44%)	33 (66%)	
Poor	28 (56%)	17 (34%)	
Sleep (categorical)			0.08
Good	22 (44%)	33 (66%)	
Worse	19 (38%)	11 (22%)	
Worst	9 (18%)	6 (12%)	
Frailty Index (a.u.)	0.24 (0.13)	0.21 (0.14)	0.26

Numbers are shown as mean (SD) or N (%). Abbreviations: SD = standard deviation; a.u. = arbitrary unit, counts = relative mean change in acceleration; kg = kilograms; m^2^ = square meter; BMI = body mass index; ART = antiretroviral therapy.

**Table 2.** Sleep quality between PLWH and controls

	Model A	Model B	
Variable	Poor vs. Good	Worse vs. Good	Worst vs. Good
PLWH (Yes)	2.88 (1.22, 6.79), 0.02	1.69 (1.06, 2.75), 0.03	1.76 (0.95, 3.45), 0.08
Sex (Female)	0.51 (0.21, 1.27), 0.15	0.77 (0.46, 1.25), 0.29	0.60 (0.30, 1.16), 0.14
Age (Year)	0.88 (0.79, 0.98), 0.02	0.91 (0.80, 1.01), 0.10	0.80 (0.66, 0.96), 0.02
Comorbidities (Any)	2.13 (0.81, 5.60), 0.13	1.20 (0.69, 2.08), 0.52	2.20 (1.11, 4.56), 0.03

Data are odds ratios (95% confidence intervals), p-value from logistic regressions. Model A is based on the dichotomized sleep score. Model B is based on the three-level categorical sleep score.

**Table 3.** Sleep quality and frailty in PLWH and controls

	Model A1	Model A2	Model B1	Model B2
***(1) PLWH***				
Intercept	0.14 (0.08, 0.20), <0.0001	0.15 (0.08, 0.22), 0.0001	0.14 (0.08, 0.19), <0.0001	0.14 (0.08, 0.21), <0.0001
Sex (Female)	0.03 (−0.03, 0.10), 0.31	0.02 (−0.05, 0.10), 0.54	0.04 (−0.03, 0.10), 0.25	0.03 (−0.04, 0.10), 0.43
Age (year)	0.01 (−0.00, 0.01), 0.06	0.01 (−0.00, 0.01), 0.21	0.01 (0.00, 0.01), 0.04	0.01 (−0.00, 0.01), 0.19
Comorbidities (Any)	−0.02 (−0.09, 0.06), 0.65	−0.02 (−0.11, 0.06), 0.61	−0.02 (−0.09, 0.05), 0.50	−0.02 (−0.10, 0.06), 0.64
Sleep (Dichotomous)			-	-
Poor	0.15 (0.09, 0.21), <0.0001	0.14 (0.07, 0.21), 0.0004	-	-
Sleep (Categorical)				
Worse	-	-	0.12 (0.06, 0.19), 0.0004	0.11 (0.04, 0.18), 0.004
Worst	-	-	0.21 (0.12, 0.30), <0.0001	0.22 (0.12, 0.33), <0.0001
CD4*	-	0.01 (−0.03, 0.05), 0.49	-	0.01 (−0.02, 0.05), 0.42
ART duration (year)	-	0.00 (−0.00, 0.01), 0.36	-	0.00 (−0.00, 0.01), 0.29
***(2) Controls***				
Intercept	0.13 (0.07, 0.20), <0.0001	-	0.14 (0.08, 0.20), <0.0001	-
Sex (Female)	0.05 (−0.03, 0.13), 0.22	-	0.05 (−0.03, 0.12), 0.19	-
Age (year)	0.01 (0.00, 0.02), 0.04	-	0.01 (0.00, 0.02), 0.02	-
Comorbidities (Any)	0.11 (0.02, 0.19), 0.01	-	0.08 (0.00, 0.16), 0.04	-
Sleep (Dichotomous)			-	
Poor	0.06 (−0.03, 0.14), 0.18	-	-	-
Sleep (Categorical)				
Worse	-	-	0.01 (−0.08, 0.10), 0.79	-
Worst	-	-	0.15 (0.03, 0.27), 0.01	-

Data are Estimate (95% confidence intervals), *p* value from regression models. Models A (A1 and A2) are based on the dichotomized sleep score. Models B (B1 and B2) are based on the three-level categorical sleep score. Models A2 and B2 are further controlled for CD4 cell counts and ART duration (thus for PLWH only).

* Results for 1-SD increase. Abbreviations: ART = antiretroviral therapy.

## Data Availability

The datasets generated during and/or analyzed during the current study are available from the corresponding author on reasonable request.
